# Supplementary material for: Online Left-Hemispheric In-Phase Frontoparietal Theta tACS Modulates Theta-Band EEG Source-Based Large-Scale Functional Network Connectivity in Patients with Schizophrenia: A Randomized, Double-Blind, Sham-Controlled Clinical Trial
Source: Biomedicines. 2023 Feb 20;11(2):630. doi: 10.3390/biomedicines11020630 (PMC9953127; doi:10.3390/biomedicines11020630)
Supplement: Supplementary file 1 [file biomedicines-11-00630-s001.zip › biomedicines-2171970-supplementary.pdf]

Supplementary materials

The supplementary materials contain:

Supplementary text, figures, tables, and references

1. Supplementary text

Randomization and Blinding

A study coordinator not involved in the execution of the trial carried out the randomization by using an online randomization tool for clinical trials (https://www.sealedenvelope.com/). Participants were assigned to the active or sham stimulation in a 1:1 ratio using blocked randomization with randomly permuted blocks of four. The allocation concealment system was performed through central randomization, in which the researcher contacted the study coordinator after enrolling and registering the participant. The allocation concealment was further ensured by the administration of theta-tACS using "study mode of the device" in which a five-digit numerical code specific to individual participants was entered into the devices (Eldith DC stimulator Plus, NeuroConn, Ilmenau, Germany) that resulted in either active or sham stimulation [1]; i.e., the researcher got the randomization code and a unique five-digit numerical code for each participant from the study coordinator while the theta-tACS administrators entered the code for study mode into the devices. The study coordinator had continuous access to the randomization list and unblinded the trial after the final visit of the last participant. Not until the trial was unblinded did the participants, theta-tACS administrators, researchers and clinical raters know the actual stimulation types. The only reason for premature code-breaking was that any suspected unexpected serious adverse reaction (SUSAR) occurred. The study coordinator would disclose the treatment code before a SUSAR was reported to the local institutional review boards (IRB) and the health agency. Once the masking code was broken, the treatment for the participant would be discontinued.

Effectiveness of blinding

Each participant was asked to answer the question of whether he or she had received active theta-tACS or sham stimulation shortly after the 1st session of stimulation and at the end of treatment. After the trial was unblinded, analyses showed that 83.3% of participants receiving active theta-tACS and 72.2% of those receiving sham guessed they had received active stimulation shortly after the 1st session of stimulation. Fisher's exact

test revealed no statistically significant between-group differences ($p = 0.69$), suggesting the satisfactory effectiveness of our blinding protocol. Similarly, active guesses between the two groups were not different at the end of treatment ($p = 0.53$)

Dropout

Dropout was considered after the absence in two consecutive theta-tACS sessions or declined consent to participate after receiving the first theta-tACS session. In this trial, 36 patients were randomly allocated to receive active online theta-tACS (n=18) or sham stimulation (n=18) (Figure S2: CONSORT Flowchart) and all of them completed 10 sessions of the trial. One participant in the active group dropped out due to withdrawal of consent after completing the 1-week follow-up visit. The current study analyzed 35 patients with complete EEG data.

Dual n-back task

Dual n-back task (Brain Workshop software v4.8.4) is a dual version n-back paradigm involving both visuospatial and auditory-verbal WM tasks and has been used for computerized WM training in healthy individuals [2] and schizophrenia patients [3]. In this task, squares at 8 different locations showed up sequentially (stimulus length, 500 ms; inter-stimulus interval, 2,500 ms) on a computer screen every 3 seconds. Simultaneously with the presentation of the squares, one of eight consonants showed up sequentially through a speaker. Participants had to judge whether the location of a square and the consonant they heard matched the one n-back before (the same n value for both visual and auditory targets). Each training session had 20 blocks (each block consisting of 20 + n trials) while each block included six auditory and six visual targets (four appearing in only one modality, and two appearing in both modalities simultaneously) whose locations were random. Participants had to make responses manually by pressing the mouse left-click button for visual targets and the right-click button for auditory targets. No responses were required for non-targets. If a target was correctly detected, a flashing green light signal would show up as positive feedback. If a target was falsely detected, a flashing blue light signal would show up as negative feedback. The dual n-back training was designed to continuously vary its difficulty by modifying the WM load (i.e., the level of n) and thereby tracking the participants' performance. Each training session began at n = 1. Participants' performance was analyzed after each block and the level of n for the next block would be adapted according to the following principle. The level of n increased by 1 in the next block if the mistakes per modality made by the participant were <3. Conversely, n decreased by 1 if the mistakes per modality made by the participant were >5. In all other cases, the n was kept unchanged. Participants came to the laboratory and took part in the WM training sessions twice daily for 5 weekdays (total of 10 sessions), with each session lasting for approximately 25 minutes. The time interval between twice-daily sessions was >3 hours. It was known that 10-session dual n-back training alone failed to significantly

improve the severity of negative symptoms in schizophrenia [3].

Online correction of eye movement and artifacts

The software module built in the Neuro Prax® TMS/tES compatible full band DC-EEG system provides real-time correction of EEG artifacts caused by blinking and eye or body movements. By choosing a correction mode of "artifact correction and correction of eye movements" from the box at the toolbar, the EEG signal was processed by a special algorithm that has been developed by the manufacturer [4] to (1) eliminate signal changes exceeding a certain threshold (standard value is 200 μV) indicating artifacts, e.g., movements of patient or cables during EEG recording, and (2) eliminate or suppress signals from the eye on the EEG without disturbing the EEG signal itself. Calibration was necessary to estimate the influence of horizontal movements, vertical movements and blink artifacts on the EEG. The calibration was done every time after applying electrodes to the patient's head. After carefully preparing the skin and applying the electrodes for EOG recording the calibration took approx. 3 minutes. An impedance check was carried out before calibration. The calibration consisted of 3 consecutive tasks. The participants were asked to: (1) move the eyes in the vertical direction with maximum deflection, (2) move the eyes in the horizontal direction with maximum deflection, and (3) open and close the eye alternatively. After completing all tasks the calibration data were calculated. The data records on the hard disk were the original data which were not influenced by the online correction. Corrected data were subsequently exported by means of the optional "Export Tool", e.g., to EDF+.

ICLabel

The detailed methods can be found in previous publications [5, 6], but the procedures are outlined here for completeness. The ICLabel classifier (an automatic EEG independent component classifer plugin for EEGLAB) is one of the current state-of-the-art EEG independent component (IC) classifier that has been shown to perform accurately and efficiently. The ICLabel classifier used in the present study comes from the final products of the ICLabel project (aiming to provide improved classifications based on the desirable qualities of an EEG IC classifier): the ICLabel classifier, dataset, and website, all of which are freely available online [5]. The architecture and training paradigm of the ICLabel classifier were selected through a cross-validated comparison between six candidate versions. A key component of the greater ICLabel project is the educational ICLabel website (accessible at https://iclabel.ucsd.edu/tutorial) which collects submitted classifications from EEG researchers around the world to label a growing subset of the ICLabel training set. The evolving ICLabel dataset of anonymized IC features can be downloaded from https://github.com/lucapton/ICLabel-Dataset [6]. To achieve accuracy across EEG recording conditions, the ICLabel dataset used to train and evaluate the ICLabel classifier encompasses a wide variety of EEG datasets from a multitude of

paradigms. These example ICs are paired with component labels collected through the ICLabel website from hundreds of contributors. Finally, to maintain sufficient computational efficiency, relatively simple IC features are used as input to an artificial neural network architecture (ANN) that, while slow to train, computes IC labels quickly. The end result is made freely and easily available through the ICLabel plug-in for the EEGLAB software environment [7, 8]. The classifier can be downloaded through the EEGLAB extensions manager under the name ICLabel or can be downloaded directly from https://github.com/sccn/ICLabel. The ICLabel classifier estimates IC classifications as compositional vectors across seven IC categories and computes IC class probabilities across these classes as described below [5]. In our study, the ICLabel was run after EEG dataset had been decomposed using independent component analysis (ICA). The IC classification information was saved to the EEG structure in the matrix. Next, the probability ranges for labeling component as artifact for rejection of non-brain ICs (i.e., muscle, eye, heart, line noise, channel noise, and other ICs) were set between 0.9 (min) to 1 (max). Those components labeled as artifact were selected and removed automatically.

Brain ICs	They contain activity believed to originate from locally synchronously activity in one (or sometimes two well-connected) cortical patches. The cortical patches are typically small and produce smoothly varying dipolar projections onto the scalp. Brain ICs tend to have power spectral densities with inversely related frequency and power and, often, exhibit increased power in frequency bands between 5 and 30 Hz.
Muscle ICs	They contain activity originating from groups of muscle motor units (MU) and contain strong high-frequency broadband activity aggregating many MU action potentials (MUAP) during muscle contractions and periods of static tension. These ICs are effectively surface EMG measures recorded using EEG electrodes. They are easily recognized by high broadband power at frequencies above 20–30 Hz. Often times they can appear dipolar like Brain ICs, but as their sources are located outside the skull, their dipolar pattern is much more localized than for brain sources.
Eye ICs	They describe activity originating from the eyes, induced by the high metabolic rate in the retina that produces an electrical dipole (positive pole at the cornea, negative at the retina). Rotating the eyes shifts the projection of this standing dipole to the frontal scalp. Eye ICs can be further subdivided into ICs accounting for activity associated with horizontal eye movements and ICs accounting for blinks and vertical eye movements. Both have scalp projections centered on the eyes and show clear quick or sustained "square" DC-shifts depending on whether the IC is describing blinks or eye movements respectively.
Heart ICs	They, though more rare, can be found in EEG recordings. They are effectively electrocardiographic (ECG) signals recorded using scalp EEG electrodes. They are recognizable by the clear QRS-complexes in their time series and often have scalp projections that closely approximate a diagonal linear gradient from left-posterior to

	right-anterior. Heart ICs can rarely have localized scalp projections if an electrode is placed directly above a superficial vein or artery.
Line noise ICs	They capture the effects of line current noise emanating from nearby electrical fixtures or poorly grounded EEG amplifiers. They are immediately recognizable by their high concentration of power at either 50 Hz or 60 Hz depending on the local standard. These effects can only be well separated if the line noise interference is spatially stationary across the EEG electrodes. Otherwise, it is unlikely that a single IC will be able to describe the line noise activity. Instead, several or even all components may be contaminated to varying degrees.
Channel noise ICs	They indicate that some portion of the signal recorded at an electrode channel is already nearly statistically independent of those from other channels. These components can be produced by high impedance at the scalp-electrode junction or physical electrode movement, and are typically an indication of poor signal quality or large artifacts affecting single channels. If an ICA decomposition is primarily comprised of this IC category, it strongly indicates that the data has received insufficient preprocessing.
Other ICs	They, rather than being an explicit category, act as a catch-all for ICs that fit none of the previous types. These primarily fall into two categories: ICs containing indeterminate noise or ICs containing multiple signals that ICA decomposition could not separate well. For ICA-decomposed high-density EEG recordings (64 channels and above), the majority of ICs typically fall into this category.

Additional information about computation of lagged phase synchronization and the statistics

Additional details on the exact low resolution electromagnetic tomography (eLORETA) and connectivity algorithm can be found in previous research [9, 10]. In brief, EEG connectivity analysis was performed using the eLORETA software. Regions of interest (ROIs) were defined using seed voxels (derived from Table S2). Connectivity between pairs of ROIs was then defined as the lagged phase synchronization (LPS, i.e., the non-linear ,non-instantaneous dependence) of intracortical EEG-source estimates. LPS computes the phase synchrony between intracortical signals in the frequency domain using normalized Fourier transforms. Therefore it is a measure of nonlinear functional connectivity. The instantaneous "zero-lag" contribution is excluded from the total phase synchronization by statistically partialing out the instantaneous component of the total connectivity to minimize the effects of associated artifacts and volume conduction, leaving only non-instantaneous synchronization. LPS measures the similarity of two time series by means of the phases of the signal after the instantaneous similarity has been removed. A value of 0 indicates no synchronization and a value of 1 indicates perfect synchronization. LPS is thought to contain only physiological connectivity information. To assess the difference in change in LPS from baseline to each postbaseline assessment between pairs of ROIs for theta

frequency band (5-7Hz) across groups (active vs. sham), eLORETA performed independent sample t-tests, thereby obtaining t-statistic images of the change in brain connectivity. For each analysis, tests were performed by eLORETA to compare the changes in all connections between 84 ROIs for theta frequency band (5-7Hz). Furthermore, to correct for multiple comparisons associated with multiple ROIs, the eLORETA implements a non-parametric randomization procedure based on "maximal statistic"[11]. The omnibus null hypothesis was rejected if at least one t value (i.e., voxel tmax) was above the critical threshold t_{crit} for p = 0.05 (corrected) determined by 5000 data randomizations.

2. Supplementary figures

	**Baseline**	**Treatment**					**End of stimulation**	**1-week follow-up**	**1-month follow-up**
		D1	D2	D3	D4	D5	D6	D12	D36
**tACS**		● ●	● ●	● ●	● ●	● ●			
**PANSS**	●						●	●	●
**EEG**	●						●	●	

Figure S1. Timeline for treatment and assessments.
Abbreviations: D, day; tACS, Online theta (6Hz) transcranial alternating current stimulation; PANSS, Positive and Negative Syndrome Scale; EEG, Electroencephalography.

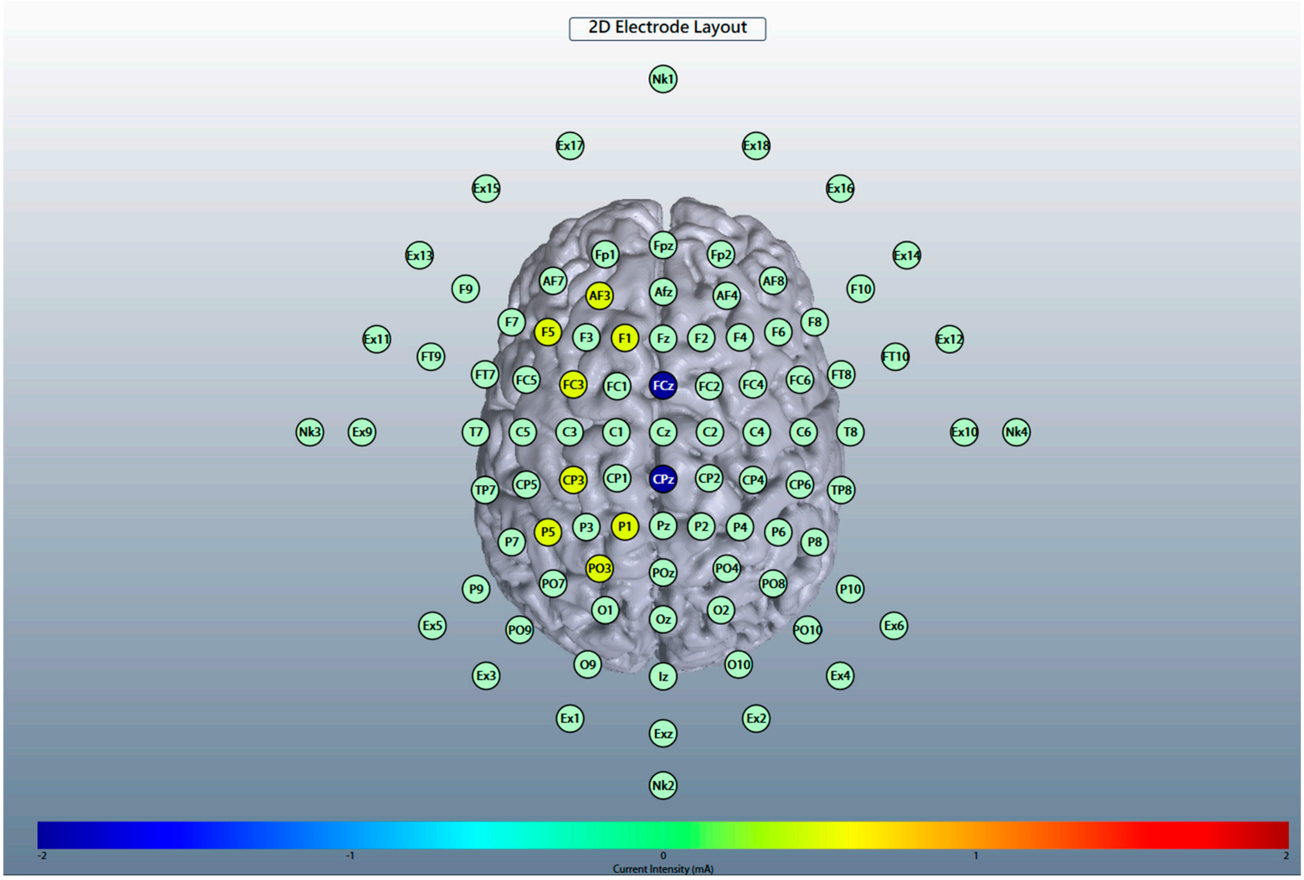

Figure S2. The 2D head model of left-hemispheric in-phase theta-rhythm frontoparietal transcranial alternating current stimulation (theta-tACS). The electrodes of the 1st DC stimulator were placed at the International 10-10 electrode positions F1, F5, AF3, and FC3 (stimulation electrodes) and CPz (return electrode). For the 2nd stimulator, the electrodes were placed at P1, P5, CP3, and PO3 (stimulation electrodes) and FCz (return electrode). A custom-made pulse generator controlled the two stimulators and created an in-phase (synchronous) setup (0° relative phase difference between the output signals of the two tACS-stimulators). The colorbar indicates the estimated current intensity that the electrodes of the stimulators receive.

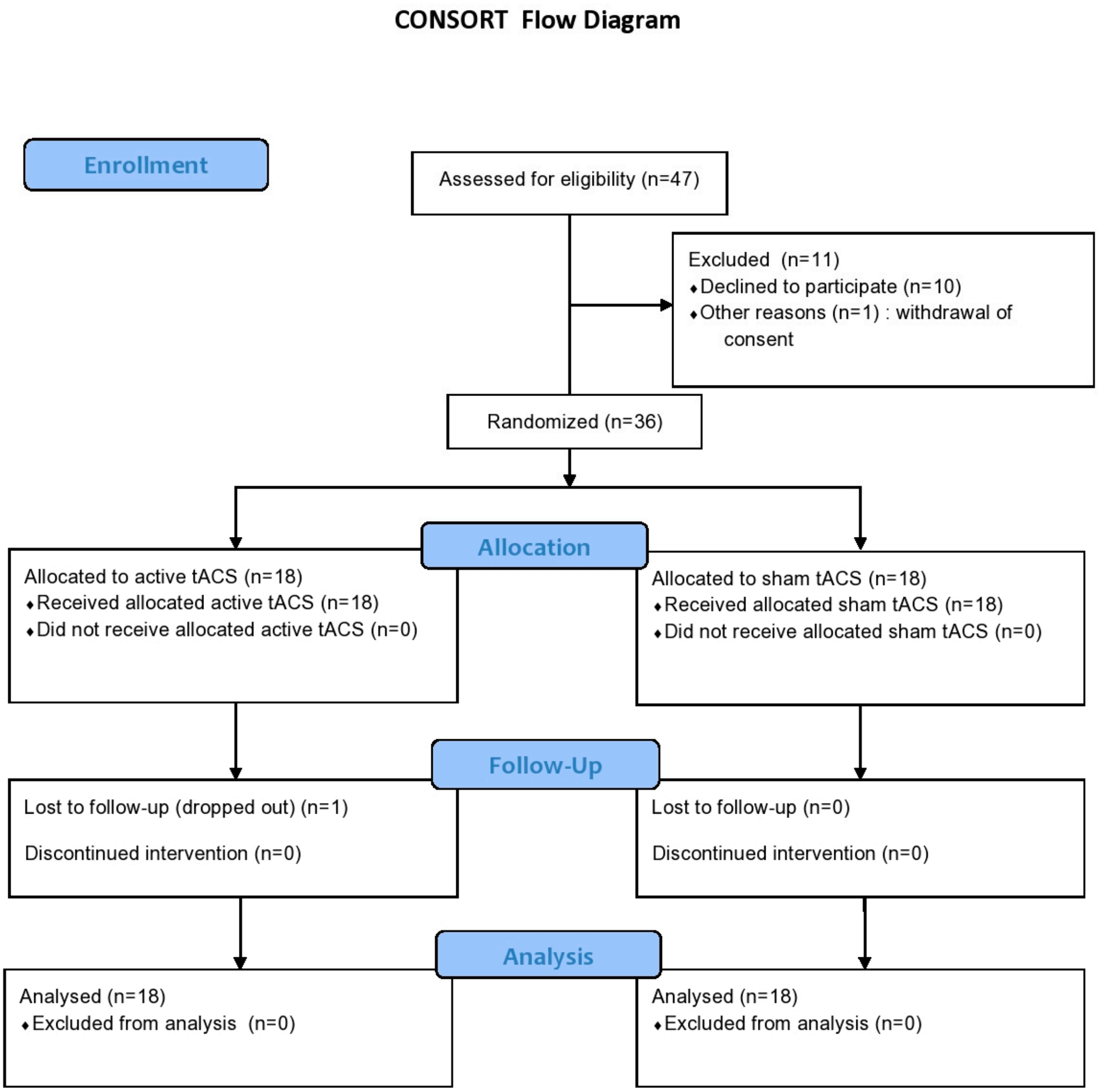

Figure S3. A CONSORT Flow Diagram of this clinical trial. In this randomized, double-blind, sham-controlled trial, patients receiving twice-daily, 2 mA, 20 min sessions of in-phase theta-tACS for 5 consecutive weekdays (n=18) or sham stimulation (n=18) were included in the intention-to-treat (ITT) analyses. Complete EEG data from the active group (n=17) and the sham group (n=18) were included in the per-protocol (PP) analyses.

3. Supplementary tables

Table S1. A CONSORT checklist of this clinical trial.

CONSORT 2010 checklist of information to include when reporting a randomised trial

Section/Topic	Item No	Checklist item	Reported on page No
**Title and abstract**			
	1a	Identification as a randomised trial in the title	1
	1b	Structured summary of trial design, methods, results, and conclusions (for specific guidance see CONSORT for abstracts)	1
**Introduction**			
Background and objectives	2a	Scientific background and explanation of rationale	2-3
	2b	Specific objectives or hypotheses	3
**Methods**			
Trial design	3a	Description of trial design (such as parallel, factorial) including allocation ratio	3-4
	3b	Important changes to methods after trial commencement (such as eligibility criteria), with reasons	Not applicable
Participants	4a	Eligibility criteria for participants	3
	4b	Settings and locations where the data were collected	3
Interventions	5	The interventions for each group with sufficient details to allow replication, including how and when they were actually administered	3-4
Outcomes	6a	Completely defined pre-specified primary and secondary outcome measures, including how and when they were assessed	4
	6b	Any changes to trial outcomes after the trial commenced, with reasons	Not applicable
Sample size	7a	How sample size was determined	Not applicable
	7b	When applicable, explanation of any interim analyses and stopping guidelines	Not applicable
Randomisation:			
Sequence generation	8a	Method used to generate the random allocation sequence	Supplementary materials
	8b	Type of randomisation; details of any restriction (such as blocking and block size)	Supplementary materials
Allocation	9	Mechanism used to implement the random allocation sequence (such as sequentially numbered containers),	Supplementar

concealment mechanism		describing any steps taken to conceal the sequence until interventions were assigned	y materials
Implementation	10	Who generated the random allocation sequence, who enrolled participants, and who assigned participants to interventions	Supplementary materials
Blinding	11a	If done, who was blinded after assignment to interventions (for example, participants, care providers, those assessing outcomes) and how	4
	11b	If relevant, description of the similarity of interventions	Not applicable
Statistical methods	12a	Statistical methods used to compare groups for primary and secondary outcomes	7
	12b	Methods for additional analyses, such as subgroup analyses and adjusted analyses	7
**Results**			
Participant flow (a diagram is strongly recommended)	13a	For each group, the numbers of participants who were randomly assigned, received intended treatment, and were analysed for the primary outcome	7
	13b	For each group, losses and exclusions after randomisation, together with reasons	Supplementary materials
Recruitment	14a	Dates defining the periods of recruitment and follow-up	3-4
	14b	Why the trial ended or was stopped	3-4
Baseline data	15	A table showing baseline demographic and clinical characteristics for each group	7-8
Numbers analysed	16	For each group, number of participants (denominator) included in each analysis and whether the analysis was by original assigned groups	7
Outcomes and estimation	17a	For each primary and secondary outcome, results for each group, and the estimated effect size and its precision (such as 95% confidence interval)	Not applicable
	17b	For binary outcomes, presentation of both absolute and relative effect sizes is recommended	Not applicable
Ancillary analyses	18	Results of any other analyses performed, including subgroup analyses and adjusted analyses, distinguishing pre-specified from exploratory	Not applicable
Harms	19	All important harms or unintended effects in each group (for specific guidance see CONSORT for harms)	Not applicable
**Discussion**			
Limitations	20	Trial limitations, addressing sources of potential bias, imprecision, and, if relevant, multiplicity of analyses	14
Generalisability	21	Generalisability (external validity, applicability) of the trial findings	Not applicable
Interpretation	22	Interpretation consistent with results, balancing benefits and harms, and considering other relevant evidence	Not applicable
**Other information**			
Registration	23	Registration number and name of trial registry	3
Protocol	24	Where the full trial protocol can be accessed, if available	3-4
Funding	25	Sources of funding and other support (such as supply of drugs), role of funders	15

Table S2. Coordinates of the 84 cortical points (Regions of interest, ROIs) used for connectivity analyses.

ROI	Structure	x	y	z	ROI	Structure	x	y	z
1	Postcentral Gyrus	-55	-25	50	43	Postcentral Gyrus	55	-25	50
2	Postcentral Gyrus	-45	-30	45	44	Inferior Parietal Lobule	50	-30	45
3	Precentral Gyrus	-35	-25	55	45	Postcentral Gyrus	40	-25	50
4	Precentral Gyrus	-35	-20	50	46	Postcentral Gyrus	35	-25	50
5	Paracentral Lobule	-15	-45	60	47	Paracentral Lobule	15	-45	60
6	Middle Frontal Gyrus	-30	-5	55	48	Middle Frontal Gyrus	30	-5	55
7	Precuneus	-20	-65	50	49	Precuneus	15	-65	50
8	Superior Frontal Gyrus	-20	30	50	50	Superior Frontal Gyrus	20	25	50
9	Middle Frontal Gyrus	-30	30	35	51	Middle Frontal Gyrus	30	30	35
10	Superior Frontal Gyrus	-25	55	5	52	Superior Frontal Gyrus	25	55	5
11	Middle Frontal Gyrus	-20	40	-15	53	Superior Frontal Gyrus	20	45	-20
12	Insula	-40	-10	10	54	Insula	40	-5	10
13	Lingual Gyrus	-10	-90	0	55	Lingual Gyrus	10	-90	0
14	Lingual Gyrus	-15	-85	0	56	Lingual Gyrus	15	-85	0
15	Cuneus	-25	-75	10	57	Cuneus	25	-75	10
16	Fusiform Gyrus	-45	-20	-30	58	Fusiform Gyrus	45	-20	-30
17	Middle Temporal Gyrus	-60	-20	-15	59	Middle Temporal Gyrus	60	-15	-15
18	Superior Temporal Gyrus	-55	-25	5	60	Superior Temporal Gyrus	55	-20	5
19	Posterior Cingulate	-5	-40	25	61	Posterior Cingulate	5	-45	25
20	Cingulate Gyrus	-5	0	35	62	Cingulate Gyrus	5	0	35
21	Medial Frontal Gyrus	-10	20	-15	63	Subcallosal Gyrus	5	15	-15
22	Parahippocampal Gyrus	-20	-35	-5	64	Parahippocampal Gyrus	20	-35	-5
23	Parahippocampal Gyrus	-20	-10	-25	65	Parahippocampal Gyrus	20	-10	-25
24	Posterior Cingulate	-5	-50	5	66	Posterior Cingulate	5	-50	5
25	Posterior Cingulate	-15	-60	5	67	Cuneus	10	-60	5
26	Precuneus	-10	-50	30	68	Precuneus	10	-50	35
27	Anterior Cingulate	-5	30	20	69	Anterior Cingulate	5	30	20
28	Anterior Cingulate	-5	20	20	70	Anterior Cingulate	0	20	20
29	Parahippocampal Gyrus	-15	0	-20	71	Parahippocampal Gyrus	15	0	-20
30	Parahippocampal Gyrus	-20	-25	-20	72	Parahippocampal Gyrus	25	-25	-20
31	Parahippocampal Gyrus	-30	-30	-25	73	Parahippocampal Gyrus	30	-25	-25
32	Fusiform Gyrus	-45	-55	-15	74	Fusiform Gyrus	45	-55	-15
33	Superior Temporal Gyrus	-40	15	-30	75	Superior Temporal Gyrus	40	15	-30
34	Middle Temporal Gyrus	-45	-65	25	76	Middle Temporal Gyrus	45	-65	25
35	Inferior Parietal Lobule	-50	-40	40	77	Inferior Parietal Lobule	50	-45	45
36	Transverse Temporal Gyrus	-45	-30	10	78	Transverse Temporal Gyrus	45	-30	10
37	Superior Temporal Gyrus	-60	-25	10	79	Superior Temporal Gyrus	65	-25	10
38	Transverse Temporal Gyrus	-60	-10	15	80	Transverse Temporal Gyrus	60	-10	15
39	Precentral Gyrus	-50	10	15	81	Precentral Gyrus	55	10	15
40	Inferior Frontal Gyrus	-50	20	15	82	Inferior Frontal Gyrus	50	20	15
41	Middle Frontal Gyrus	-45	35	20	83	Middle Frontal Gyrus	45	35	20
42	Inferior Frontal Gyrus	-30	25	-15	84	Inferior Frontal Gyrus	30	25	-15

4. References

1. Palm, U., et al., *Evaluation of sham transcranial direct current stimulation for randomized, placebo-controlled clinical trials.* Brain Stimul, 2013. **6**(4): p. 690-5.
2. Jaeggi, S.M., et al., *Improving fluid intelligence with training on working memory.* Proc Natl Acad Sci U S A, 2008. **105**(19): p. 6829-33.
3. Li, X., et al., *The remediation effects of working memory training in schizophrenia patients with prominent negative symptoms.* Cogn Neuropsychiatry, 2019. **24**(6): p. 434-453.
4. Schlegelmilch, F., et al., *Online ocular artifact removal for dc-EEG-signals: Estimation of dc-level*, B. Technik, Editor. 2004, Ergänzungsband 2., Band. p. 340-341.
5. Pion-Tonachini, L., K. Kreutz-Delgado, and S. Makeig, *ICLabel: An automated electroencephalographic independent component classifier, dataset, and website.* Neuroimage, 2019. **198**: p. 181-197.
6. Pion-Tonachini, L., K. Kreutz-Delgado, and S. Makeig, *The ICLabel dataset of electroencephalographic (EEG) independent component (IC) features.* Data Brief, 2019. **25**: p. 104101.
7. Delorme, A. and S. Makeig, *EEGLAB: an open source toolbox for analysis of single-trial EEG dynamics including independent component analysis.* J Neurosci Methods, 2004. **134**(1): p. 9-21.
8. Delorme, A., et al., *EEGLAB, SIFT, NFT, BCILAB, and ERICA: new tools for advanced EEG processing.* Comput Intell Neurosci, 2011. **2011**: p. 130714.
9. Pascual-Marqui, R.D., et al., *Assessing interactions in the brain with exact low-resolution electromagnetic tomography.* Philos Trans A Math Phys Eng Sci, 2011. **369**(1952): p. 3768-84.
10. Whitton, A.E., et al., *Electroencephalography Source Functional Connectivity Reveals Abnormal High-Frequency Communication Among Large-Scale Functional Networks in Depression.* Biol Psychiatry Cogn Neurosci Neuroimaging, 2018. **3**(1): p. 50-58.
11. Nichols, T.E. and A.P. Holmes, *Nonparametric permutation tests for functional neuroimaging: a primer with examples.* Hum Brain Mapp, 2002. **15**(1): p. 1-25.
